# Synthesis of Biocompatible Silver-Doped Carbonate Hydroxyapatite Nanoparticles Using Microwave-Assisted Precipitation and In Vitro Studies for the Prevention of Peri-Implantitis

**DOI:** 10.3390/jfb14070385

**Published:** 2023-07-21

**Authors:** Saifuddin Aziz, Ika Dewi Ana, Yusril Yusuf, Harno Dwi Pranowo

**Affiliations:** 1 Department of Chemistry, Faculty of Mathematics and Natural Sciences, Universitas Gadjah Mada, Yogyakarta 55281, Indonesia; saifuddin.a@mail.ugm.ac.id; 2 Department of Dental Biomedical Sciences, Faculty of Dentistry, Universitas Gadjah Mada, Yogyakarta 55281, Indonesia; ikadewiana@ugm.ac.id; 3 Research Collaboration Center for Biomedical Scaffolds National Research and Innovation Agency of the Republic of Indonesia (BRIN) and Universitas Gadjah Mada (UGM), Bulaksumur, Yogyakarta 55281, Indonesia; yusril@ugm.ac.id; 4 Department of Physics, Faculty of Mathematics and Natural Sciences, Universitas Gadjah Mada, Yogyakarta 55281, Indonesia

**Keywords:** antibacterial, biocompatible, hydroxyapatite, peri-implantitis, silver

## Abstract

A carbonate-hydroxyapatite-based antibacterial implant material with low cytotoxicity was synthesized. The silver ion (Ag^+^) was incorporated into CHA material, resulting in silver-doped carbonate hydroxyapatite (CHA-Ag). The microwave-assisted precipitation method was used to synthesize the CHA-Ag material. The amount of Ag^+^ was varied at 0.005, 0.010, and 0.015 mol fractions ($\chi_{Ag}$). The XRD results showed that the diffractograms corresponded with hydroxyapatite (ICSD 98-05-1414), without any additional phase. The presence of carbonate ions was indicated by vibrations at wavenumber of 871, 1411, and 1466 cm^−1^ in the infrared spectra. The CHA-Ag materials were agglomerates of nanosized particles with low crystallinity. The particle size and crystallinity of the materials decreased due to the incorporation of CO_3_^2−^ and Ag^+^. The incorporated Ag^+^ successfully inhibited peri-implant-associated bacterial growth. The antibacterial ability increased alongside the increase in the Ag^+^ amount. The pre-osteoblast MC3T3E1 cell could grow up to >70% in the MTT assay, despite the use of Ag^+^ as a dopant. The cell viability was higher in the CHA-Ag-containing media than in the CHA-containing media. The MTT assay also revealed that the CHA-Ag cytotoxicity decreased even though the Ag^+^ amount increased. The CHA-Ag-15 had the lowest cytotoxicity and highest antibacterial activity. Therefore, the optimal amount of Ag^+^ in the CHA-Ag formulation was $\chi_{Ag}$ = 0.015.

## 1. Introduction

Hydroxyapatite is widely used as a dental implant coating material due to its osseointegration ability [1,2]. The dental implant appears to be a safe treatment method. However, dental implant materials’ lack of antibacterial properties triggers peri-implantitis infection [3]. Peri-implantitis is an implant-related bacterial infection around the dental implant [4,5,6]. This bacterial infection causes bone resorption and implant loss, possibly requiring implant removal [7,8,9,10]. Mechanical decontamination, laser treatment, or either treatment in combination with antiseptics and antibiotics is used for peri-implantitis treatment. Achieving the therapeutic concentration at the target site requires a high antibiotic dosage [11]. However, antibiotic use causes resistance that renders one more susceptible to peri-implantitis in the future [12]. There are three stages in the bacterial infection processes: adhesion, biofilm formation, and maturation [13]. The adhesion and biofilm stages have important roles in the bacterial infection process. Bacterial adhesion is the initial stage of bacterial infection [14]. Then, in the biofilm stage, the bacteria are resistant to antibodies, phagocytes, and antibacterial drugs [4]. Therefore, inhibition of the bacterial adhesion process is necessary to reduce the potential for peri-implantitis.

Metal ions are an ideal choice for inhibiting bacterial adhesion due to their broad-spectrum and long-term antibacterial effect [4,13]. Ag^+^ is the metal ion most frequently used as an antibacterial agent [15]. In the hydroxyapatite (HA) implant material, Ag^+^ can be incorporated via Ca^2+^ substitution [16]. However, Ag^+^ has a cytotoxicity effect in addition to its antibacterial properties. The use of Ag^+^ with a mol fraction ($\chi_{Ag}$) > 0.03 in hydroxyapatite inhibited fibroblast cell growth [12]. Therefore, it should be restricted below a 0.03 mol fraction to minimize the cytotoxicity effect. Previously, researchers have shown that using a Ag^+^ < 0.03 mol fraction can inhibit bacterial growth with a low cytotoxicity effect [17,18]. However, this has never been attempted for peri-implant-associated bacteria.

While researchers have succeeded in minimizing the toxicity of metal-ion-doped implants, improving their biocompatibility remains challenging. The implant material becomes meaningless if it has antibacterial activity but a low tissue integration ability [4]. Material–tissue integration capabilities also play important roles in implant-associated bacterial infection. For an implant’s success, material–tissue integration must occur before bacterial adhesion [13]. The osseointegration ability of hydroxyapatite should be improved to obtain faster material–tissue integration. Carbonate (CO_3_^2−^)-doped hydroxyapatite (CHA) has better in vivo osseointegration than HA due to its lower crystallinity [19]. Another way to improve the osseointegration of HA is nanometer-size formation [20].

The HA nanoparticle can be synthesized through wet chemical precipitation by mixing a calcium- and phosphate-containing solution at pH > 7. However, the wet precipitation method requires a long synthesis time. Zuo et al. [21] and Xu et al. [22] applied the wet chemical precipitation method for hydroxyapatite nanoparticle synthesis, requiring 1 and 1.5 h of stirring, and then continued with the aging process. Kolmas et al. [23] used a 48 h aging time to obtain CHA-Ag material using wet chemical precipitation. Wet chemical precipitation can be combined with a microwave heating system to overcome the long synthesis process.

This work aimed to produce a hydroxyapatite-based antibacterial implant material with minimal toxicity and enhanced bioactivity. The silver-doped carbonate hydroxyapatite (CHA-Ag) nanoparticle was synthesized with Ag^+^ below a 0.03 mol fraction. The CHA-Ag was synthesized using a microwave-assisted precipitation method to obtain a nanosized particle. The nanosized carbonate hydroxyapatite was chosen due to its improved bioactivity. The Ag^+^ below a 0.03 mol fraction and carbonate hydroxyapatite were expected to provide antibacterial properties to a hydroxyapatite-based implant material with minimal cytotoxicity that can inhibit peri-implant-associated bacterial growth.

## 2. Materials and Methods

### 2.1. Materials

The precursors used for synthesis—CaNO_3_·4H_2_O, (NH_4_)_2_HPO_4_, Na_2_CO_3_, and AgNO_3_—were purchased from Merck (USA). The peri-implant-associated bacteria for the antibacterial test were *Aggregatibacter actinomycetemcomitans, Fusobacterium nucleatum*, *Porphyromonas gingivalis*, *Prevotella intermedia*, and *Staphylococcus aureus*, which were obtained from the Research Center of the Faculty of Dentistry, Universitas Airlangga, Surabaya, Indonesia. The bacteria culture and antibacterial test media were Brain Heart Infusion (Oxoid, UK) broth and Muller–Hinton media. The cells for the MTT assay were pre-osteoblast MC3T3E1 cells from the European Collection of Authenticated Cell Cultures (ECACC). The media for the cell culture was MEM-α media, supplemented with 10% Fetal Bovine Serum, 2% Penicillin–Streptomycin, and 0.5% Fungizone (Gibco™). Trypsin EDTA 0.25% (Gibco™) was also used in the MTT assay.

### 2.2. Methods

#### 2.2.1. Materials Synthesis

The synthesis process was conducted using the microwave-assisted precipitation method in the Biomaterials Laboratory, Department of Physics, Universitas Gadjah Mada, Indonesia. The stoichiometry method of Singh et al. [12] and Safarzadeh et al. [24] was used in this synthesis process, shown in Table 1. The CaNO_3_.4H_2_O and AgNO_3_ were dissolved in distilled water with $\chi_{Ag}$ of 0.005, 0.010, and 0.015. The (NH_4_)_2_HPO_4_ and Na_2_CO_3_ were dissolved in another vessel with the P/(Ca + Ag) and C/P mol ratios of 0.6 and 1. Each solution was adjusted to a 9.85 pH using NH_4_OH. The PO_4_^3−^-CO_3_^2−^ solutions were added to the Ca^2+^-Ag^+^ solution and stirred for 10 min. Then, the suspensions were heated with a microwave oven (Sharp R728 K, 900 watts) using 30% power for 9 min. After heating, the CHA-Ag suspensions were filtered and dried at 100 °C for 6 h. The hydroxyapatite (HA) was also synthesized to compare the physicochemical properties.

#### 2.2.2. Material Characterizations

The synthesized materials were characterized using a Fourier transform infrared spectrophotometer (FT-IR), X-ray diffractometer (XRD), and electron microscope. The FT-IR (Shimadzu Prestige 21) was used to record the functional group vibrations of the synthesized materials. The XRD (Bruker D8 ADVANCE ECO) with Cu Kα radiation was used to record the X-ray diffraction patterns. The diffractograms were processed using HighScore Plus software to determine the profile-fitted peak position and its full width at half maximum (FWHM). The crystallinity and crystallite size of the material were evaluated using the crystallinity index (*CI*) and Scherrer equation:

(1) $$CI={\left(\frac{0.24}{\beta_{002}}\right)}^{3}\;\mathrm{and}\;L=\frac{K\times \lambda }{\beta_{002}\times \cos\theta }$$

where *β*_002_ is the FWHM of the 002-lattice plane, *K* is the Scherrer constant with a value of 0.89, and *λ* is 1.5406 Å for the Cu Kα [25]. The lattice parameters were estimated using the following equation [26]:

(2) $$\frac{1}{d^{2}}=\frac{4\left(h^{2}+hk+k^{2}\right)}{3a^{2}}+\frac{l^{2}}{c^{2}}$$

The morphology and atomic composition of the materials were analyzed using a transmission electron microscope (TEM, JEOL JEM-1400) and scanning electron microscope (SEM, JEOL JSM-6510LA).

#### 2.2.3. In Vitro Studies for Antibacterial Properties and Cytotoxicity

The in vitro antibacterial studies were conducted at the Research Center of the Faculty of Dentistry, Universitas Airlangga, Indonesia, using the agar well diffusion method. Antibacterial tests were carried out for the CHA and CHA-Ag samples with four repetitions. A total of 0.1 g of pelletized sample for each CHA and CHA-Ag material was sterilized using ethylene oxide gas. On the other hand, the peri-implant-associated bacteria were cultured in Brain Heart Infusion broth media and spread onto Muller–Hinton media. Then, the CHA and CHA-Ag pellets were diffused in bacteria-containing Muller–Hinton media and incubated at 37 °C for 48 h. The antibacterial activity was assessed in terms of the inhibition zone diameter.

The in vitro cytotoxicity test was conducted at the Integrated Research and Testing Laboratory, Universitas Gadjah Mada, Indonesia, using the MTT assay. The MTT assay was carried out for the CHA and CHA-Ag samples without repetition. At first, pre-osteoblast MC3T3E1 cells were cultured in MEM-α media, supplemented with 10% Fetal Bovine Serum, 2% Penicillin–Streptomycin, and 0.5% Fungizone. The cells were harvested after 80% of the cells were confluent. Then, the cells were removed from the flask using trypsin EDTA 0.25% and transferred to a sterilized conical vessel containing 1 mL of supplemented MEM-α media. Then, 10 μL of cell suspension was pipetted into a hemocytometer for cell counting. The MTT assay was initiated by cultivating the pre-osteoblast MC3T3E1 cells in the 96-well plate, followed by incubation for 24 h. Next, CHA and CHA-Ag suspensions with 4000 μL/mL concentrations were added to each well, with the volume adjusted to form a serial concentration of 23.44–3000 μL/mL, and then incubated for 48 h. Next, 100 μL/well of 0.5 mg/mL MTT assay reagent (Biobasic, United States of America) was added to each well and incubated for 4 h. DMSO was added 100 μL/well to dissolve the formazan salt. The solution’s optical density (*OD*) was measured using a Tecan Spark^®^ spectrophotometer at 570 nm. The cell viability was calculated using the following equation:

(3) $$Cell\;viability=\frac{OD\;of\;the\;treatment-OD\;control\;of\;media}{OD\;control\;of\;cells-OD\;control\;of\;media}\times 100\%$$

The cell viability of each material concentration was plotted with the log [material] vs. cell viability curve. Then, the curve was fitted with non-linear regression to calculate the IC_50_ value.

#### 2.2.4. Statistical Method

The statistical method used to help us to interpret the antibacterial test result was one-way analysis of variance (ANOVA). The results of the inhibition zone diameter were grouped based on the bacteria and material. First, ANOVA was carried out in the group of bacteria and material. In the group of bacteria, ANOVA was performed to study the effect of the Ag^+^ amounts on the bacteria. Then, ANOVA for the group of materials was performed to examine the different bacteria’s responses to Ag^+^. The post hoc Tukey test was also performed to study the significant differences between group members. Before the ANOVA was conducted, the inhibition zone diameter results for the group of bacteria and material were tested for their homogeneity and normality.

## 3. Results and Discussion

### 3.1. Material Characterization

**Figure 1:**
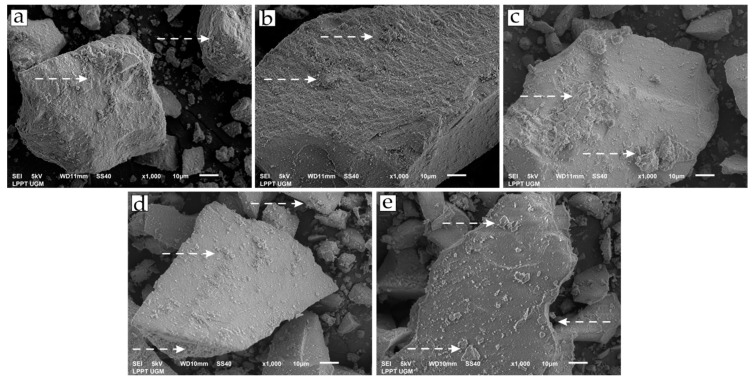
Morphology of the (**a**) HA, (**b**) CHA, (**c**) CHA-Ag-05, (**d**) CHA-Ag-10, and (**e**) CHA-Ag-15 materials.

**Figure 2:**
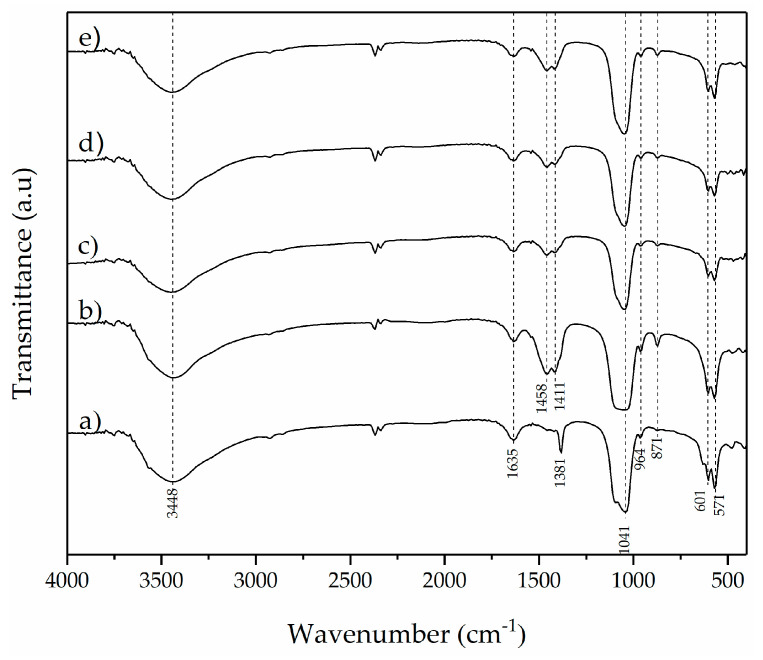
Spectra images of (**a**) HA, (**b**) CHA, (**c**) CHA-Ag-05, (**d**) CHA-Ag-10, and (**e**) CHA-Ag-15.

**Figure 3:**
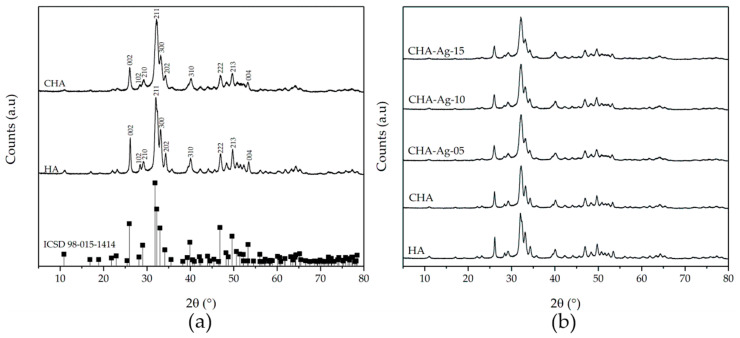
The X-ray diffractogram of the synthesized HA and CHA compared to (**a**) ICSD HA 98-015-1414 and (**b**) CHA-Ag with variations of Ag^+^.

The CHA and CHA-Ag were synthesized using the microwave-assisted precipitation method. The material characterization was carried out to validate the CHA and CHA-Ag materials’ formation and study their characteristics. The SEM images in Figure 1 show the appearance and surface morphology of the materials. The materials have the appearance of solid micro-sized materials with a rough surface. The arrow in Figure 1 shows the rough surface of the materials. There were no differences in the morphology or appearance of the synthesized materials. All materials were composed of Ca, P, and O atoms, as shown in the EDX results in Table 2. However, the Na atom appeared in the CHA and CHA-Ag materials due to the use of Na_2_CO_3_ as a CO_3_^2−^ source. The Ag atom was undetected in the EDX analysis, because the amount was less than that of the Ca, P, and Na. Figure 2 shows the vibrations of the functional groups in the materials. The vibrations at 565 and 600 ($v_{4}$; doubly degenerated bending mode), 964 ($v_{1}$; symmetric stretching), 1057 cm^−1^ ($v_{3}$; triply degenerated asymmetric stretching) confirmed the presence of the PO_4_^3−^ group in the materials [19]. The appearance of 871 ($v_{2}$; bending), 1411 ($v_{1}$; stretching), and 1466 cm^−1^ ($v_{3}$; stretching) vibrations confirmed the existence of CO_3_^2−^ ions [23,24]. The broad absorption peaks at 1600–1700 and 3200–3600 cm^−1^ belonged to the absorbed H_2_O molecules in the crystal structure [27]. The presence of the Ca atom and PO_4_^3−^ functional group proved that the materials belonged to the calcium phosphate family. Figure 3 shows that the materials had the hydroxyapatite lattice structure, as their diffraction patterns were well-matched with the hydroxyapatite (ICSD 98-05-1414) phase. However, the Ca/P ratio ranged from 1.39 to 1.55. The calcium phosphate family, with a Ca/P ratio range from 1.33–1.67, are categorized as calcium-deficient hydroxyapatite (CDHA) or precipitated hydroxyapatite (PHA) [28,29,30]. The conformity of the diffraction pattern with the hydroxyapatite phase and the presence of carbonate absorption peaks proved that the materials were carbonate hydroxyapatite.

Table 3 shows the crystal parameters of the materials. The CHA and CHA-Ag materials had almost the same lattice constant as HA. Changes in the value of the lattice constant were due to the incorporation of Ag^+^ and CO_3_^2−^ into the HA lattice. The lattice constants of the materials underwent varying changes. The Ag^+^ increased both the a- and c-axes of the hydroxyapatite lattice. Meanwhile, the CO_3_^2−^ decreased the a-axis and increased the c-axis of the hydroxyapatite lattice. The decrease in the a-axis and increase in the c-axis due to the incorporation of CO_3_^2−^ into the HA lattice are characteristic of the type-B CHA [31]. This change in the lattice constant was due to the larger PO_4_^3−^ (2.38 Å) being replaced by the smaller CO_3_^2−^ (1.76 Å) [24]. The shape and orientation of the CO_3_^2−^ also affected the lattice constant of the HA. Kubota et al. [32] revealed that the trigonal planar CO_3_^2−^ occupied the side of the tetragonal PO_4_^3−^ parallel to the c-axis. With this shape and orientation, the incorporated CO_3_^2−^ would enlarge the c-axis and reduce the a-axis of the HA lattice. The lattice constant of the materials increased when the Ag^+^ was incorporated into the HA lattice. Comparing the radii of the Ag^+^ and Ca^2+^, the Ag^+^ (1.28 Å) was larger than the Ca^2+^ (0.99 Å). Therefore, the HA lattice expanded as Ag^+^ replaced Ca^2+^ [33,34,35,36].

**Figure 4:**
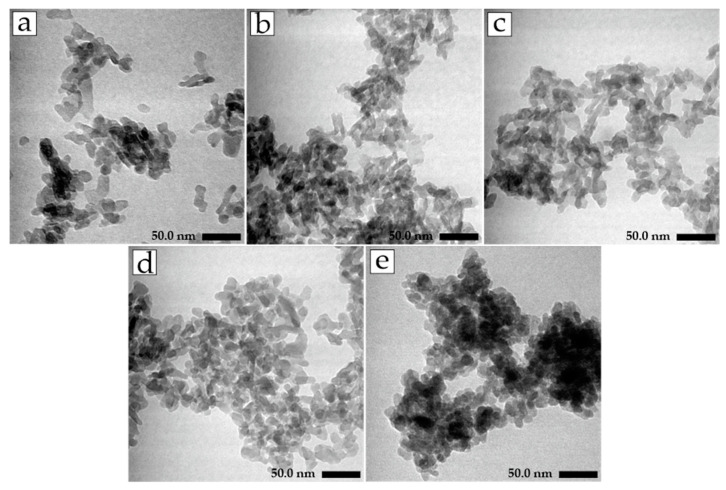
TEM images of (**a**) HA, (**b**) CHA, (**c**) CHA-Ag-05, (**d**) CHA-Ag-10, and (**e**) CHA-Ag-15.

The crystallinity, crystallite, and particle size of the materials in Table 3 also changed due to the incorporation of Ag^+^ and CO_3_^2−^. The HA material had the highest crystallinity. The crystallinity decreased when the Ag^+^ and CO_3_^2−^ were incorporated into HA lattice. The crystallite size of the materials was 17–28 nm and categorized as nanocrystalline materials [37,38]. The crystallite size calculation results were similar to the particle size measurement results using TEM. The TEM images in Figure 4 show that the materials had elongated shapes with an irregular surface morphology [39]. The lengths of HA, CHA, CHA-Ag-05, CHA-Ag-10, and CHA-Ag-15 particles based on the TEM images were 24.88, 16.48, 16.55, 18.90, and 15.82, respectively. Both the crystallite and particle sizes decreased as Ag^+^ and CO_3_^2−^ were incorporated into the hydroxyapatite lattice; however, the crystallite and particle sizes increased as the amount of Ag^+^ increased.

These results follow those of previous studies related to Ag^+^- and CO_3_^2−^-doped hydroxyapatite [40,41,42,43,44]. Both Ag^+^ and CO_3_^2−^ could inhibit the growth of HA crystals [42,44]. The substitution of the larger tetragonal PO_4_^3−^ with the smaller planar CO_3_^2−^ led to a decrease in the crystallite size of the HA [45]. The presence of CO_3_^2−^ ions in the HA lattice also decreased the surface energy [46]. Deymier et al. [46] revealed that the change in the crystal energetics controlled the crystallite size, i.e., the more CO_3_^2−^ was incorporated, the smaller the crystal was. Meanwhile, there were two perspectives regarding the effect of the Ag^+^ ion on the HA. The presence of Ag^+^ could increase the crystallite size of the HA due to the higher Ag^+^ radius (1.28 Å) compared to that of the Ca^2+^ (0.99 Å) [33,34,35,47]. Other studies reported that incorporating Ag^+^ into the HA lattice decreased the crystallite size due to the distortion of the HA lattice [43,44,48]. Substituting Ca^2+^ with Ag^+^ introduced different ionic radii into the HA lattice, resulting in the HA lattice’s distortion and crystal growth inhibition [44]. Karunakaran et al. [26] also stated that HA crystal growth inhibition was due to the potent electrostatic interaction between Ag^+^ and PO_4_^3−^. In this work, both perspectives can be observed in Table 3. The smaller crystallite size of CHA-Ag, as compared to CHA, indicated that Ag^+^ inhibited crystal growth. At the same time, the crystallite size of the CHA-Ag increased with the increase inf Ag^+^ due to the higher Ag^+^ radius as compared to that of the Ca^2+^.

### 3.2. Antibacterial Activity

**Figure 5:**
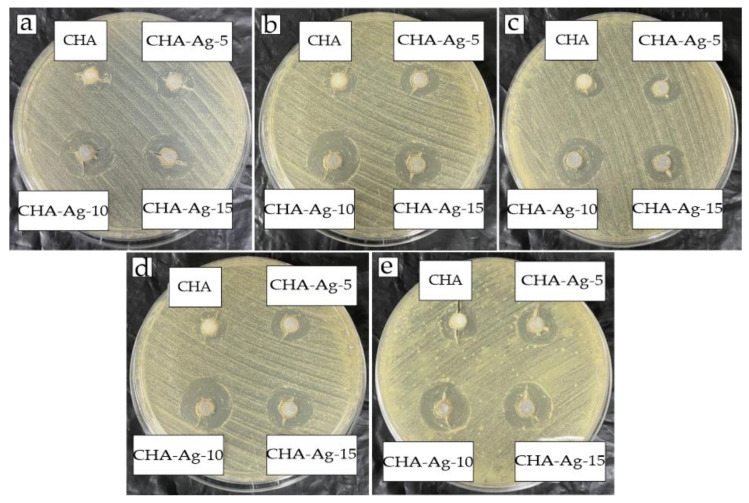
Inhibition zones of (**a**) Aggregatobacter actynomycetemcomitans, (**b**) Fusobacterium nucleatum, (**c**) Porphyromonas gingivalis, (**d**) Prevotella intermedia, and (**e**) Staphylococcus aureus.

In this work, Ag^+^ was used as a dopant in the CHA materials to provide antibacterial properties. The usage of Ag^+^ was limited to below a 0.03 mol fraction. An antibacterial test was conducted to determine whether Ag^+^ below a 0.03 mol fraction had antibacterial properties. Figure 5 shows that the synthesized materials had antibacterial activity. The synthesized materials could inhibit the growth of peri-implantitis bacteria and produce an inhibition zone. The diameter of the inhibition zone indicated the strength of the antibacterial properties of the material. The CHA-Ag materials had significantly different inhibition zone diameters compared to the CHA. Due to the oligodynamic effect, a small number of Ag^+^ ions in the CHA-Ag materials could inhibit peri-implant-associated bacteria growth [49,50]. Ag^+^ also offered more remarkable antibacterial ability than that observed in our previous studies using egg-white-modified CHA with an inhibition zone diameter of only 10.01–13.33 mm [51].

**Figure 6:**
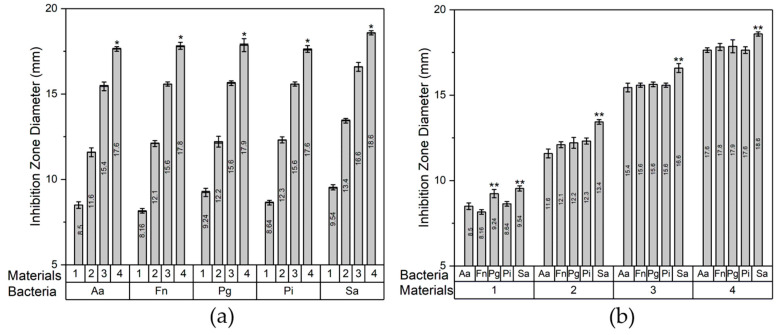
Inhibition zone diameters grouped for the (**a**) bacteria and (**b**) materials: (1) CHA, (2) CHA-Ag-05, (3) CHA-Ag-10, and (4) CHA-Ag-15. * The highest subset in the bacteria group. ** The highest subset in the materials group.

The inhibition zone diameter data were analyzed for variance in the groups of bacteria and materials. The ANOVA and post hoc Tukey test results are shown in Table 4 and Figure 6, respectively. The ANOVA results for the bacterial groups had a significant *p* < 0.05, with CHA-Ag-15 being the highest subset in the post hoc test. The results showed a difference in the inhibition zone diameter for the different amounts of Ag^+^, and the CHA-Ag-15 had the most increased antibacterial activity. In the group of materials, the ANOVA result also had a significant value of *p* < 0.05. This result indicated that the different bacteria responded differently to the same material. The post hoc Tukey results showed that the inhibition zone diameter of *Staphylococcus aureus* was the widest among all the bacteria. It was concluded that Staphylococcus aureus was the bacteria most affected by Ag^+^. *Staphylococcus aureus* ps a Gram-positive bacterium, while the others are Gram-negative. Gram-positive bacteria only have a thick peptidoglycan in the cell wall [52]. Meanwhile, Gram-negative bacteria have a lower permeability outer membrane that serves as a solid barrier for the cell [53,54]. The low permeability of the outer membrane makes Gram-negative bacteria more difficult for Ag^+^ to penetrate than Gram-positive bacteria [54].

### 3.3. Cytotoxicity Evaluation

Ag^+^ is a broad-spectrum antibacterial agent and a toxic material for mammalian cells, including fibroblast and osteoblast cells [9,21,22,55]. In addition, the incorporated Ag^+^ contributes to hydroxyapatite toxicity. Therefore, a cytotoxicity test for CHA-Ag materials was necessary. The pre-osteoblast cell was selected for the CHA-Ag toxicity test because of its role in bone formation [56]. The cytotoxicity of the CHA-Ag materials was determined using the MTT method. The pre-osteoblast cells were incubated for 48 h with various concentrations of CHA and CHA-Ag ranging from 23.44 to 3000 μg/mL. The living cells reduced the MTT reagent to purple formazan salt. The amount of formazan equaled the number of living cells and was expressed as cell viability [57].

**Figure 7:**
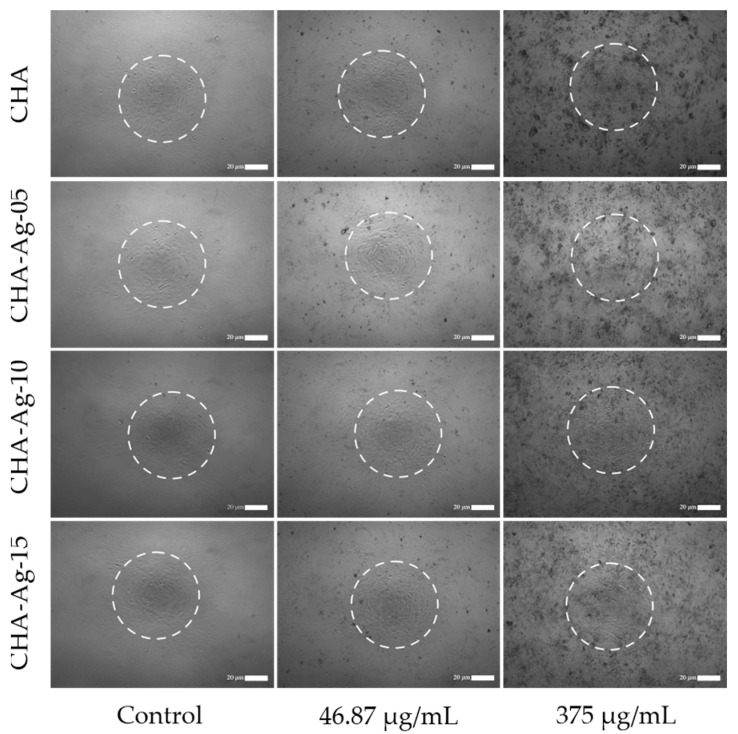
The microscopic image of pre-osteoblast MC3T3E1 cells in the media-containing materials.

**Figure 8:**
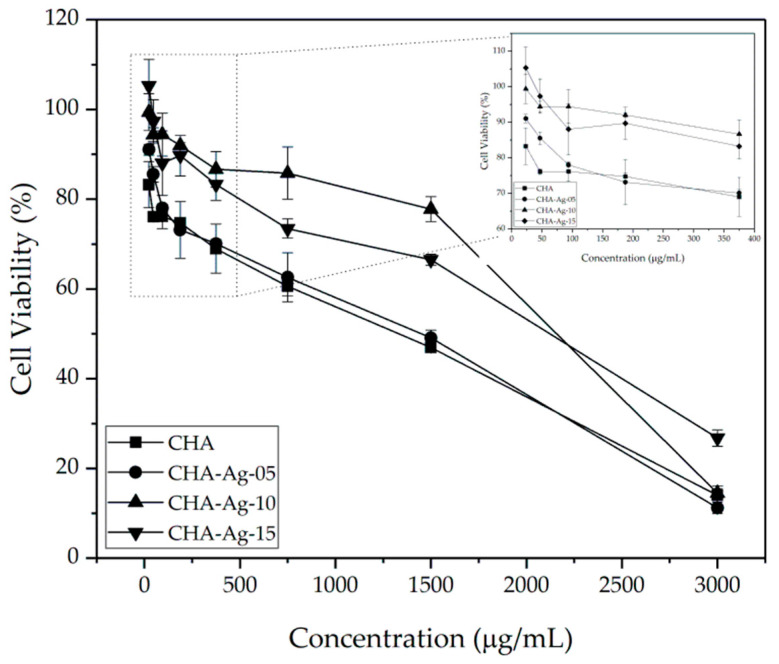
The pre-osteoblast MC3T3E1 cell viability with varying material concentrations.

The microscopic image in Figure 7 shows pre-osteoblast MC3T3E1 cells grown in media-containing materials. The white circles were the pre-osteoblast MC3T3E1 cells, while the black patches were material particles. The pre-osteoblast cell viabilities shown in Figure 8 were above 50%, but the cell viabilities reduced as the concentration of the materials increased. The decrease in cell viability occurred in all the CHA materials. This proved that the presence of Ag^+^ did not cause reductions in cell viability. These results differ from the antibacterial activity shown in Figure 6, which was affected by the amount of Ag^+^. As shown in Figure 7, increasing the material concentration showed an increase in the undissolved particles in the cell growth medium. The increase in the undissolved particles was accompanied by a decrease in cell viability, as shown in Figure 8. The presence of undissolved materials was also supported by the fact that the solubility of HA was 43.56 μg/mL [58]. The materials were undissolved when the concentration exceeded 43.56 μg/mL. Therefore, it was concluded that the undissolved particles of the synthesized materials reduced cell viability.

**Figure 9:**
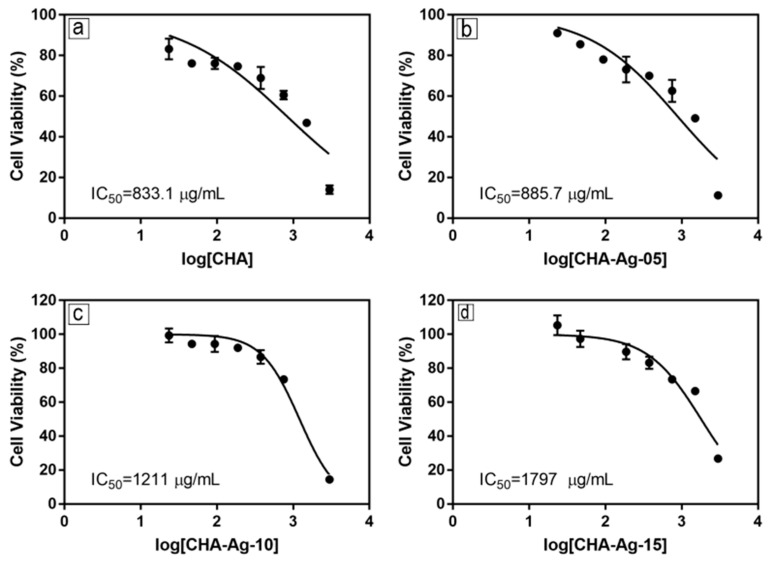
Pre-osteoblast cell viability as a function of (**a**) CHA, (**b**) CHA-Ag-05, (**c**) CHA-Ag-10, and (**d**) CHA-Ag-15 concentrations.

Then, the cell viability data were used to calculate the IC_50_ value. The IC_50_ in Figure 9 shows that the IC_50_ of the CHA-Ag materials was higher than that of the CHA. This result means the CHA-Ag materials were less toxic or more biocompatible than the CHA. In Figure 8, the CHA-Ag materials had better cell viability than the CHA. The cell viability of CHA-Ag was higher than that of CHA. The viability of the pre-osteoblast MC3T3E1 cells in this work was not affected by the presence of Ag^+^. However, the cell viability of the pre-osteoblast MC3T3E1 was affected by the undissolved particles of the materials. The increase in the number of undissolved particles due to the increased material concentration decreased cell viability. Because cell viability was not affected by the presence of Ag^+^, a comparison of the physical properties of CHA and CHA-Ag was necessary. The physical properties of the materials are shown in Table 3. The CHA-Ag materials had lower crystallinity and smaller crystallite and particle sizes than the CHA. Table 3 and Figure 9 show that CHA-Ag had a smaller size and lower crystallinity than CHA, but the cell viability of CHA-Ag was higher than that of CHA. Therefore, the smaller and less crystalline CHA-Ag was less cytotoxic or more biocompatible than the larger and more crystalline CHA.

Webster et al. [59] and Shi et al. [60] had similar results. Webster et al. [59] found that metal-doped HA had higher cell viability than non-doped HA. The higher cell viability in the metal-doped HA was related to the grain size of the materials. The metal-doped HA had a smaller grain size than the non-doped HA. The smaller grain size of the metal-doped HA might contribute to cell adhesion enhancement. Shi et al. [60] compared the osteoblast-like cell activity with different sizes of hydroxyapatite materials. The osteoblast-like cell had better proliferation activity towards low crystalline hydroxyapatite with a 20 nm particle size than the highly crystalline 80 and 200–500 nm material, because the smaller, low crystalline HA could more easily to penetrate into cells and stimulate cell growth. Since this work only involved in vitro studies, it will be necessary to conduct in vivo studies on the effects of particle size or the low amount of silver-doped CHA in future studies.

## 4. Conclusions

Carbonate hydroxyapatite (CHA-Ag) nanoparticles were successfully synthesized using microwave-assisted precipitation. The materials were agglomerates of 16.72–23.47 nm nanoparticles. The incorporated Ag^+^ and CO_3_^2−^ decreased the materials’ crystallinity, as well as the crystallite and particle sizes. The presence of Ag^+^ in the CHA-Ag successfully inhibited peri-implant-associated bacterial growth. The antibacterial effect was directly proportional to the Ag^+^ amount. CHA-Ag with a 0.015 mol fraction of Ag^+^ (CHA-Ag-15) had the highest antibacterial ability. Although doped with Ag^+^ at 0.005, 0.010, and 0.015 mol fractions ($\chi_{Ag}$), CHA-Ag had a pre-osteoblast MC3T3E1 cell viability > 70%. The CHA-Ag materials also had lower cytotoxicity or better biocompatibility, as their IC_50_ value was higher than the IC_50_ of the CHA material. The highest IC_50_ value belonged to CHA-Ag-15 material. Therefore, the optimal amount of Ag^+^ as a CHA dopant for obtaining the highest antibacterial properties but the lowest cytotoxicity was a 0.015 mol fraction of Ag^+^ $\left(\frac{Ag}{Ca+Ag}\right)$.

## Figures and Tables

**Table 1:** The stoichiometry of CHA-Ag synthesis.

Materials	$\left(\chi_{Ag}\right)$	Stoichiometry			
		Ca(NO_3_)_2_	AgNO_3_	(NH_4_)_2_HPO_4_	Na_2_CO_3_
HA	-	10	-	6	-
CHA	-	10	-	6	6
CHA-Ag-05	0.005	9.95	0.05	6	6
CHA-Ag-10	0.010	9.90	0.10	6	6
CHA-Ag-14	0.015	9.85	0.15	6	6

**Table 2:** The elemental analysis results using EDX.

Materials	Element (%Mass)				Ca/P
	Ca	P	O	Na	
HA	27.40	15.20	48.74	-	1.39
CHA	28.51	14.91	45.32	1.25	1.48
CHA-Ag-05	25.59	13.65	48.53	1.38	1.45
CHA-Ag-10	27.99	15.08	45.62	1.33	1.43
CHA-Ag-15	34.41	16.68	40.56	0.92	1.59

**Table 3:** Crystal parameters and particle size of the materials.

Materials	Lattice Constant		Crystallinity (%)	Crystallite Size (nm)	Particle Size (nm) ^1^
	a	c			
HA	9.358	6.837	63.3	28.29	23.47
CHA	9.339	6.854	43.6	25.00	21.42
CHA-Ag-05	9.344	6.866	15.0	17.49	16.98
CHA-Ag-10	9.350	6.867	15.9	17.86	16.55
CHA-Ag-15	9.349	6.864	16.4	18.02	16.72

^1^ TEM images measurement.

**Table 4:** The results of ANOVA for the groups of bacteria and materials.

In the Group of	Significance		
	Normality Test	Homogeneity Test	ANOVA
Bacteria	>0.05	>0.05	0.00
Materials	>0.05	>0.05	0.00

## Data Availability

The data presented in this study are available on request from the corresponding author.
